# Computed tomography based radiomic signature as predictive of survival and local control after stereotactic body radiation therapy in pancreatic carcinoma

**DOI:** 10.1371/journal.pone.0210758

**Published:** 2019-01-18

**Authors:** Luca Cozzi, Tiziana Comito, Antonella Fogliata, Ciro Franzese, Davide Franceschini, Cristiana Bonifacio, Angelo Tozzi, Lucia Di Brina, Elena Clerici, Stefano Tomatis, Giacomo Reggiori, Francesca Lobefalo, Antonella Stravato, Pietro Mancosu, Alessandro Zerbi, Martina Sollini, Margarita Kirienko, Arturo Chiti, Marta Scorsetti

**Affiliations:** 1 Department of Biomedical Sciences, Humanitas University, Pieve Emanuele (Milan), Italy; 2 Radiotherapy and Radiosurgery, Humanitas Clinical and Research Center, Rozzano (Milan), Italy; 3 Diagnostic Radiology, Humanitas Clinical and Research Center, Rozzano (Milan), Italy; 4 Pancreatic Surgery, Humanitas Clinical and Research Center, Rozzano (Milan), Italy; 5 Nuclear Medicine, Humanitas Clinical and Research Center, Rozzano (Milan), Italy; North Shore Long Island Jewish Health System, UNITED STATES

## Abstract

**Purpose:**

To appraise the ability of a radiomics signature to predict clinical outcome after stereotactic body radiation therapy (SBRT) for pancreas carcinoma.

**Methods:**

A cohort of 100 patients was included in this retrospective, single institution analysis. Radiomics texture features were extracted from computed tomography (CT) images obtained for the clinical target volume. The cohort of patients was randomly divided into two separate groups for the training (60 patients) and validation (40 patients). Cox regression models were built to predict overall survival and local control. The significant predictors at univariate analysis were included in a multivariate model. The quality of the models was appraised by means of area under the curve and concordance index.

**Results:**

A clinical-radiomic signature associated with Overall Survival (OS) was found significant in both training and validation sets (p = 0.01 and 0.05 and concordance index 0.73 and 0.75 respectively). Similarly, a signature was found for Local Control (LC) with p = 0.007 and 0.004 and concordance index 0.69 and 0.75. In the low risk group, the median OS and LC in the validation group were 14.4 and 28.6 months while in the high-risk group were 9.0 and 17.5 months respectively.

**Conclusion:**

A CT based radiomic signature was identified which correlate with OS and LC after SBRT and allowed to identify low and high-risk groups of patients.

## Introduction

Patients affected by pancreatic adenocarcinoma have a typically un-favourable prognosis, with a 5-year overall survival (OS) rate as low as 6% [1]. Surgery, as a treatment of choice, leads to 5-year OS rates of about 20 to 25%. Nevertheless, a large fraction of the total patients, are unfit to surgery already at diagnosis (due to the stage of the disease or other concomitant exclusion criteria) [2]. For this reason, chemo-radiotherapy is a frequently adopted solution for many patients but with the drawback of some significant rate of severe toxicity (grade 3 to 4) and a still low rate of survival. The median OS is in the range of 5 to 15 months and the 2-year OS is about 30% [3]. The role of stereotactic body radiotherapy (SBRT) was investigated [4], but only few trials were published with reports about significant late toxicity rates [5–11]. Our institutional experience [12] demonstrated that SBRT with a fractionation of 45Gy in 6 sessions is an effective and safe therapeutic option for non-operable as well as for isolated local recurrences with a median OS of 13 months for the non-operable cases. 1 and 2 year OS were 59±7% and 18±9% respectively. In the same study freedom from local progression (FFLP) was 87±6% at 2 years [12]. The comparative assessment of the clinical use of SBRT in the management of pancreatic cancer demonstrated (Table 5 in that original publication), with a quite variable range of fractionation regimens, very consistent findings in terms of FFLP and median OS times ranging from 8 to 20 months, again consistent with our findings [12].

In this context, it would be advisable to introduce in the clinical practice, methods and tools suitable to predict the probability of tumor control and/or of survival enabling a better treatment personalization and to stratify patients in risk classes. The quantitative analysis of textural featuers (radiomics) of the tumor tissues from the quantification of various imaging sets might contribute to the definition of these tools. Advanced computational methodologies could enable the identification of quantitative predictive descriptors (signatures) of biological features cancer tissues [13–14].

Limited literature is available on the use of radiomics for pancreas cancer. Yue [15] stratified patients in various risk groups using pre- and post-radiotherapy positron emission tomography and computed tomography (PET, CT, PET/CT) images in a small group of 26 patients and determined the value of texture features with respect to response to treatment. The authors concluded that texture analysis of metabolic imaging is feasible and might contribute positively to the outcome prediction.

Hanania [16] investigated CT data to assess degree of malignancy of intraductal papillary mucinous neoplasms (IPMNs) for 53 patients and found that high-grade IPMNs can be significantly distinguished from lower grade disease.

Permuth [17], by means of a retrospective analysis on 38 patients with surgically-resected and pathologically-confirmed IPMNs assessed the capability of ‘radiomic’ CT features with respect to standard radiological methods to identify predict IPMN lesions.

Chen [18] assessed the response during chemoradiation therapy by means of radiomic analysis of daily CT images in a group of 20 patients. Authors found that the CT based radiomic features underwent significant changes during the course of therapy, with larger variations observed in the patients with good response to therapy.

Eilaghi [19] investigated the role of CT texture features with respect to overall survival in pancreatic ductal adenocarcinoma on 30 patients finding a signature of five features statistically associated to survival.

Our experience with hepatocellular carcinoma [20] and lung cancer [21] suggested the possibility to derive predictive models from the use of limited number of textural features computed from radiotherapy planning CT scans. The objective of the present study is to appraise the role of radiomics features computed from CT images to correlate with local control and overall survival in patients treated with SBRT for inoperable pancreatic cancer. The aim was therefore to identify, if existing, a purely radiomics based signature suitable to stratify the patients in outcome related risk groups. The inclusion of further clinical factors (like the other treatments or other known risk factors) might further strengthen the signature but was out of the scope of this first investigation.

## Materials and methods

### Study design

This is a retrospective investigation performed on 100 patients treated in one single institute. The retrospective study was approved by the Humanitas Cancer Center Ethics Committee by written notification. The ethical committee waived the requirement of further written informed consent for this retrospective, non clinical, study. All data were fully anonymized prior to access and use. All patients had histologically proven locally advanced pancreatic cancer and were treated with SBRT with a prescription dose of 45Gy in 6 fractions. Inclusion criteria were, as described in the original study [12]: Histologically-proven inoperable primary pancreatic adenocarcinoma, Age ≥18 years, Karnofsky Performance score of at least 70, lesions with maximum diameter not exceeding 5cm, negative lymph-node, absence of distant metastasis. Exclusion criteria were: previous abdominal RT, nodal and/or metastatic disease, gastric or duodenal obstruction, and concurrent chemotherapy. For all patients, complementarily to the textural features and the outcome data, some clinical parameters such as the age, the sex, the various chemotherapy regimens and the volume of lesions treated with SBRT were recorded.

Treatment details can be found in our original clinical report [12]. In summary, SBRT treatments were delivered by means of Volumetric Modulated Arc Therapy (VMAT) in its RapidArc with 6MV flattening filter free photon beams generated by a TrueBeam linear accelerator (Varian Medical Systems, Palo Alto, USA). Two partial arcs were used as a class solution for all the patients. Treatment planning and dose calculation was performed using the Eclipse planning system, version 11 (Varian Medical Systems, Palo Alto, USA) For all patients, the planning aim was to achieve a target coverage of V_95%_ = 100% for the clinical target volume (where V_x%_ is the percentage volume covered by the x% isodose). The dose–volume constraints for the OARs were duodenum D_1cm3_ < 36 Gy (where D_xcm3_ is the dose received by x cm^3^ of the structure), stomach and small bowels D_3cm3_ < 36Gy, kidneys V_15Gy_ < 35%, liver, total spared volume (Vtot _ V_21Gy_,) > 700 cm^3^, and spinal cord D_1cm3_ < 18Gy.

All patients underwent a pretreatment contrast-free planning CT imaging and the clinical target volume contoured on these images for the radiation treatment was used as the input for the textural analysis. The segmentation was pre-processed to exclude from the contours the presence of vessels or biliary stent, calcifications of any potential artifact. The entire processing of the target volumes was performed by a team of senior radiation oncologist, radiologist and physicists and consensus volumes were used for this study.

### Textural radiomics analysis

All the textural features were computed using the LifeX software tools [22]. A total of 41 features were derived from the CT images and grouped according to intensity, shape and second and higher order features [20]. In particular the following families of featuers were extracted: the gray-level co-occurrence matrix (GLCM); the neighborhood gray-level different matrix (NGLDM); the grey level run length matrix (GLRLM) and the grey level zone length matrix (GLZLM). A detailed description of all the features, can be found in [23]. From the histogram of the gray level distribution in the volume, the following features were extracted: the minimum, maximum, mean and standard deviation of the hounsfield units (HU) distribution as well as the skewness, the kurtosis, the entropy and the energy derived form this distribution [24]. The shape of the volumes was quantified by means of the spericity and the compacity. In the S1 Table from the supplementary materials to this article we detailed the list of the features computed and used for the analysis. Images were sampled to symmetrical voxels of 2 mm. A HU binning was applied resampling the images in intervals of 10 HU.

### Statistical analysis

The statistical analysis of the data was performed by means of dedicated routines based on the open source R platform (version 3.3) [25]. The entire cohort of patients was randomly split in two sub-cohorts of 60 and 40 patients respectively. These two groups were used for the training and validation phases of the analysis. In the training phase, all the radiomic features, as well as the clinical predictors age and sex, were investigated for their prognostic value relatively to overall survival and local control (LC) with univariate Cox regression. Optimal separation thresholds were determined per each predictor. These were chosen in correspondance of the minimum of the p-value distribution obtained from a running threshold from the Wilcoxon test. The Pearson’s correlation among the pre-selected features was determined and the correlated features were identified and excluded from further analysis. The feature’s prognostic value was assessed by means of the concordance index [26].

Multivariate Cox regression was then performed either keeping all the significant features (model A) or including in the model only the un-correlated ones (model B) as input for the backward elimination phase. This strategy was applied to both OS and LC analysis and therefore four models were built: A-OS, B-OS (and similarly for LC). Elastic net regularization, dealing with multiple cross-related variables, was used to select the most significant covariates [20]. The elastic net regularization does automatic variable selection eliminating groups of correlated variables allowing to identify the best predictors when a set is larger than the number of cases. Calibration was evaluated with Hosmer and Lemen test [20]. The data of both training and validation sets were split into low- (below threshold) and high- risk (above threshold) groups by the median of the Cox’s prediction. The multivariate models were verified on the independent validation set.

## Results

The main characteristics of the cohort of 100 patients are summarized in Table 1. The mean volume of the target was 24.59±17.6 cm^3^ (range: 2.8–109.5 cm^3^). Concerning the SBRT treatment, all patients respected the planning aims for target coverage. Table 2 summarizes the data for the training and validation sets.

**Table 1:** Summary of patient characteristics.

Parameter	Number of patients (and %)
Number of patients	100
Age (years)	Mean: 70.5 Range: 41–91
Sex	Males: 47 Females: 53
Tumor site:	Head: 65 (65%) Body/Tail 35 (35%)
Stage	Locally advanced: 98 (98%) Borderline Resectable 2 (2%) Patients with detectable CA19-9 (>1.5U/ml): 72 (72%)
Chemotherapy before SBRT	Yes: 55 (55%) No: 45 (45%)
Response after chemotherapy	Partial response: 31 (56%) Stable disease: 17 (31%) Progressive disease: 7 (13%)

**Table 2:** Summary of the patients’ demographics for the training and validation cohorts.

	Training	Validation
Number of cases	60	40
Sex:	Male: 28 (47%) Female: 32 (53%)	Male: 19 (48%) Female: 21 (52%)
Age [years]	Median: 71.0 Range: 41–90	Median: 70.5 Range: 48–91
Target Volume [cm^3^]	Mean: 23.2±14.1 Range: 2.8–92.8	Mean: 27.0±18.8 Range: 3.9–109.5

**Fig 1:**
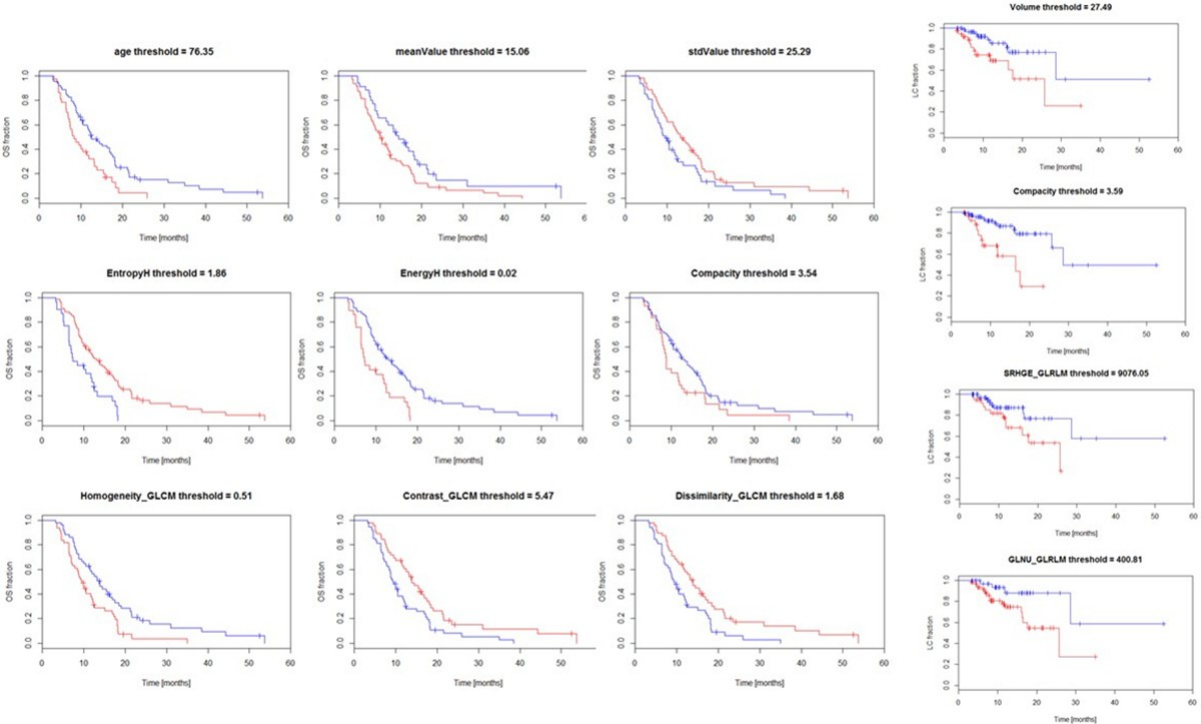
Overall survival (OS) and local control (LC) curves stratified according to the best threshold for the clinical and radiomic features found to be significant at univariate analysis. Data are shown for the training dataset. In the figures the blue lines correspond to the stratum above the threshold.

From the analysis of clinical and textural data, 9 predictors resulted significant at univariate test for OS and 4 for LC. Table 3 summarizes the relative findings while Fig 1 illustrates the OS (and LC) graphs stratified according to the nine and four, respectively, significant predictors for the training cohort.

**Table 3:** List of the clinical and radiomics features found to be significant at univariate analysis.

Feature name	threshold	p-value	Concordance index
Overall Survival			
Age	76.4	0.002	0.66
MeanValue	15.1	0.02	0.66
StdValue	25.3	0.04	0.64
EntropyH	1.9	<0.001	0.69
EnergyH	0.02	<0.001	0.70
Compacity	3.5	0.05	0.63
Homogeneity_GLCM	0.5	0.004	0.67
Contrast_GLCM	5.5	0.009	0.65
Dissimilarity_GLCM	1.7	0.005	0.66
Local Control			
Volume	27.5	0.02	0.62
Compacity	3.6	<0.001	0.63
SRHGE_GLRLM	9076.0	0.04	0.65
GLNU_GLRLM	400.8	0.02	0.63

Concerning the model A-OS, of the 9 input predictors, only 4 were retained as significant(the mean value (meanValue) and the Homogeneity_GLCM as significant with p<0.01 and the standard deviation value (stdValue) as well as the Dissimilarity_GLCM with p<0.1). The area under the curve (AUC) from the receiver operating characteristic (ROC) curve built out of the model resulted 0.81 (95% confidence interval (95%CI): 0.64–0.98) for the training set and 0.73 (95%CI: 0.54–0.92) for the validation set. The concordance index for this model was 0.74±0.03 in the training and 0.72±0.03 in the validation.

In the case of the model B-OS, only two variables resulted significant (Age and Homogeneity_GLCM with p<0.01), the AUC was 0.80 (95%CI: 0.66–0.94) in the training and of 0.73 (95%CI: 0.53–0.93) in the validation. The concordance index for this model was 0.73±0.03 in the training and 0.75±0.03 in the validation.

Concerning Local Control, the model A-LC retained 2 features (Volume and grey level non-uniformity (GLNU_GLRM) with p<0.01). The AUC was 0.72 (95%CI: 0.55–0.88) for the training and 0.70 (95%CI: 0.52–0.89) for the validation. The concordance index was 0.69±0.08 for training and 0.75±0.10 for validation. The model B-LC provided similar results with 2 retained features (short run low grey level emphasis and grey level non-uniformity). The AUC was 0.65 (95%CI: 0.52–0.81) for the training and 0.61 (95%CI: 0.50–0.78) for the validation. The concordance index was 0.70±0.07 for training and 0.73±0.09 for validation.

**Fig 2:**
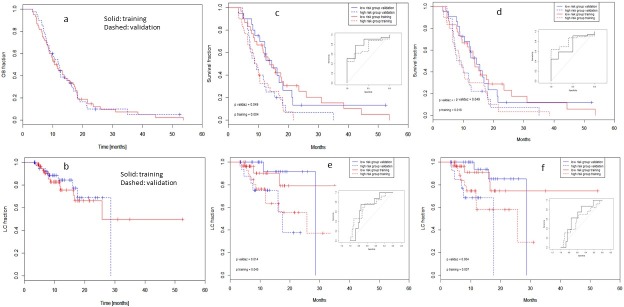
a) and b): Overall survival (OS) and Local Control (LC) curves for the training (solid red line) and validation (blue dashed line) cohorts of patients without any stratification; c) and d): Overall survival (OS) curves for the multivariate models A and B respectively. The sub-panels represent the ROC curves built out of the models. e) and f): Local Control (LC) curves as for the above. In the survival curves, solid lines correspond to the low risk group of patients; blue lines to the validation (red for the training) set. In the ROC curves, solid line is for the training, dashed for the validation.

Fig 2A and Fig 2B show the OS and LC for the two cohorts in the training and validation sets. The median OS for the entire cohort was: 11.6 months (CI95%:9.6–13.9); the median LC was: 28.6 months (CI95%: 25.7–32.1). Fig 2 shows also the result of the multivariate Cox model built for Overall Survival and Local Control. OS from the Model A-OS is shown in panel (c) and the corresponding ROC curves as an internal sub-panel for the training and validation sets. In the survival curves, solid lines correspond to the low risk group of patients; blue lines to the validation (red for the training) set. In the ROC curves, solid line is for the training, dashed for the validation. Panel (d) reports the same OS data for the Cox model B-OS built using in input only the set of uncorrelated variables. The p-values from the Wilcoxon test comparison between high and low risk groups resulted 0.004 and 0.05 in the training and validation cohorts for the models A-OS and B-OS. Panel (e) reports the LC curves from Model A-LC and panel (f) from Model B-LC with the same conventions as above.

Table 4 summarizes the median times and the 95% confidence interval for OS and LC derived from the two models A and B.

**Table 4:** Median and 95% confidence interval for the overall survival time and for the local control time.

	Training		Validaz	
	Low risk	High risk	Low risk	High risk
	Model A-OS, Overall Survival			
Median	13.8	9.2	14.4	9.0
CI95%	11.4–23.5	7.0–13.9	12.2–21.5	7.2–15.4
	Model B-OS, Overall Survival			
Median	13.8	8.8	14.4	8.6
CI95%	114–23.5	7.0–16.8	12.2–21.2	7.0–18.0
	Model A-LC, Local Control			
Median	Not reached	27.5	28.6	17.5
CI95%	Not reached	11.8-not reached	17.5-not reached	16.0-not reached
	Model B-LC, Local Control			
Median	Not reached	Not reached	28.6	17.5
CI95%	25.7	11.8-not reached	12.5-Not reached	7.62-not reached

**Fig 3:**
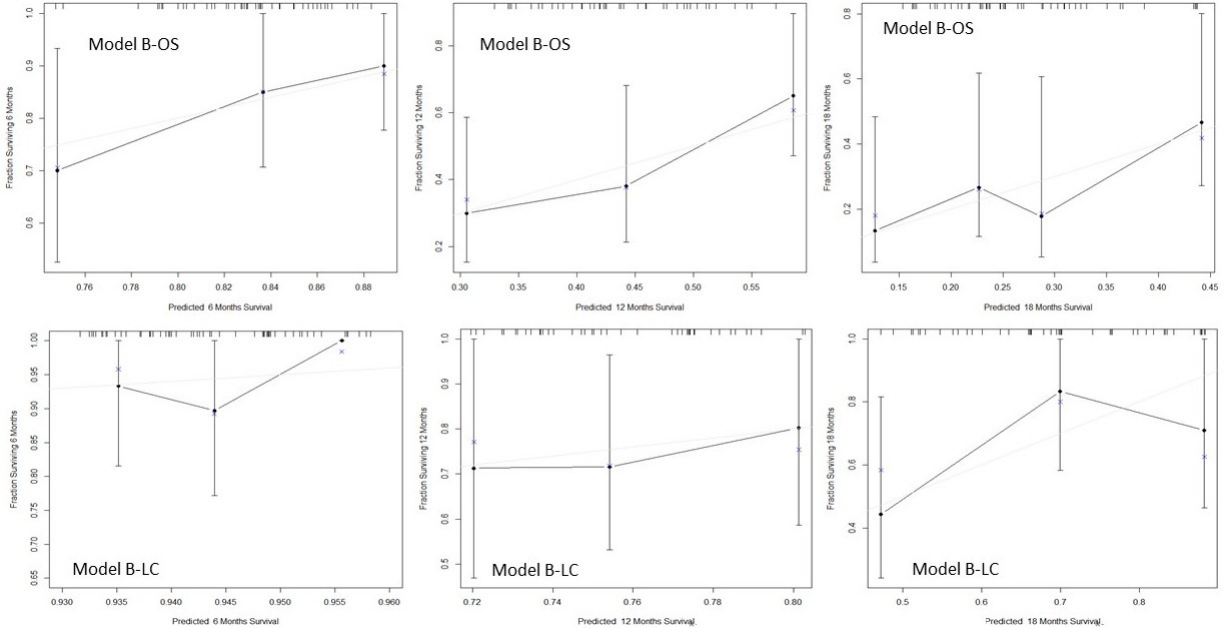
Calibration plots at 6,12 and 18 months for the Cox models B for overall survival and local control.

Fig 3 shows the calibration plots at 6,12 and 18 months for the models B-OS and B-LC. Considering these graphs, the best survival and local control estimates were achieved at 6 and 12 months while at 18 months the deviation of the estimated from the observed values becomes more relevant due to the paucity of events and the limited sample.

## Discussion

This study aimed to appraise the correlation between some radiomic signatures and the clinical outcome in a retrospective analysis of 100 patients with pancreas cancer treated according the institutional protocols. As noticed, textural analysis has been rarely applied to pancreatic cancer and almost no efforts, so far, were put in the radiomic assessment of outcome data [17–20] and none in association to SBRT. In this study, planning CT scans were used as a basis of the analysis to derive the textural features which demonstrated the possibility to be modelled vs OS and LC. The methodology adopted in the present study is derived from similar other investigations [27–28] and aims to simplicity. The panel of features available for testing did not included higher order texture (e.g. wavelets). Our hypothesis was that a radiomics signature, if existing, should be found within the set of most robust and easy to compute classes of features. As discussed in [20], the use of conventional planning CT and the clinical target volumes (with minor pre-processing edits) is another factor of simplicity which can guarantee a straightforward implementation of the radiomic methodology in the clinical practice. Although final scope of a “predictive” tool is the classification of patients in risk groups (or any similar stratification) prior to therapy, in practice, the models has to be trained and tuned retrospectively on cohorts of patients where the outcome of treatments is known. In this perspective, our investigation covers the first elements of the entire chain, i.e. the determination of a (potentially) useful signature, in a retrospective investigation on a cohort of patients completely treated (with radiotherapy and/or chemotherapy). Further prospective studies shall be designed to validate the predictive models on un-treated cohort of patients to measure their reliability and performance. Another limiting factor in the study was the exclusion, from the predictive model, of any clinical factor which might have influenced the outcome. In practice, all patients received the same SBRT treatment but some different chemotherapy regimen. The inclusion of clinical factors might strengthen the predictive value of the predictive models but, in the present study, the aim was to identify, if existing, a purely radiomic feature. Further studies will be devoted to the refinement of the models and the elimination (or explicit inclusion) of all the valuable factors. Concerning chemotherapy, 55% of the patients received it prior to SBRT. These pre-treated patients were evaluated after the end of chemotherapy with Torax-abdominal CT scan with the evidence of partial response (56%), stable disease (31%) or local progression limited to pancreatic cancer (13%). No patient showed distant progression of disease, therefore all patients were eligible for SBRT.

The study was performed by evaluating only one random split of the cohort into training and validation sets (60–40% split). We shall acknowledge that this procedure might have some inherent selection bias due to the single evaluation. Multiple repetition of the random split could have been introduced as a mitigation factor but, if this seems to be relatively easy in a simple classification experiment, the procedure creates some methodological problem when combining multiple actuarial curves. We decided to stick on the single split which could be of course seen as a reasonable simulation of a typical prospective study. Increasing significantly the sample size would have been the other option.

Unfortunately, the consistency of the cohort of patients in the current study is limited due to the relative paucity of patients with advanced pancreatic cancer treated with SBRT. This is also acknowledged as a limit of the study. This is in particular true given the need to split the total sample into validation and training, and the further stratification in low- and high-risk groups limits the number of events per class of patients. Longer follow up and larger cohorts would be needed to improve this aspect.

Nevertheless, the data available allowed to identify radiomics signatures with two features for both OS and LC and to identify the low- and high- risk groups significantly separated at the median of the predictions. A search for an optimal threshold did not lead to significant improvements and was not considered as robust and as simple as the median. Despite we performed an independent validation on our own data, further assessment of the robustness of the thresholds should be performed by enlarging the population under investigation, possibly to data from other centers.

## Conclusions

A radiomics signature made of simple clinical and textural features allowed to generate a predictive model for OS and LC in patients affected by advanced pancreatic cancer treated with SBRT. A fair discrimination power was found applying the model to training and validatin samples. Further validation studies would be advisable to confirm these findings.

## Supporting information

S1 Table An overview of the meaning of all the radiomic features used for the study as defined in the LifeX package [ref 22 of the main article]. For the explicit mathematical definitions readers are referred to ref 22 and further references therein.
(DOCX)
