# Supplementary material for: Computed tomography based radiomic signature as predictive of survival and local control after stereotactic body radiation therapy in pancreatic carcinoma
Source: PLoS One. 2019 Jan 18;14(1):e0210758. doi: 10.1371/journal.pone.0210758 (PMC6338357; doi:10.1371/journal.pone.0210758)
Supplement: S1 Table — For the explicit mathematical definitions readers are referred to ref 22 and further references therein. (DOCX) [file pone.0210758.s001.docx]

**Computed tomography based Radiomic Signature as predictive of survival and local control after stereotactic body radiation therapy in pancreatic carcinoma**

**SUPPLEMENTARY MATERIALS**

**S1 Table** An overview of the meaning of all the Radiomic Features used for the study as defined in the LifeX package [ref 22 of the main article]. For the explicit mathematical definitions readers are referred to ref 22 and further references therein.

| **FIRST ORDER FEATURES**  **From the histogram of the gray level distribution in the volume, the following features were extracted:**  The min value (minValue): minimum HU in the volume analyzed  The max value (maxValue): maximum HU in the volume analyzed  The mean value (meanValue): mean HU in the volume analyzed  The standard deviation (stdValue): standard deviation of the HU in the volume analyzed  The skewness: measures the asymmetry of the grey level distributions in the histogram  The kurtosis: measures the shape of the grey level distribution (peaked or flat) relative to a normal distribution.  The entropy: measures the randomness of the distribution  The energy: measures the uniformity of the distributions  **From the geometrical analysis of the volumes:**  The Sphericity: measures how spherical a volume is. Sphericity=1 is for a perfect sphere.  The Compacity: measures the degree of compactness of a volume.  **HIGHER ORDER FEATURES**  **From the gray-level co-occurrence matrix (GLCM):** The GLCM matrix takes into account the arrangement of pairs of voxels to calculate the indexes.  Homogeneity: the homogeneity of the grey level voxel pairs  Energy: the uniformity of gray level voxel pairs  Contract: also called variance or inertia, is the local variation in the GLCM  Correlation: is the linear dependency of the grey levels in the GLCM  Entropy: is the randomness of the grey level voxel pairs  Dissimilarity: is the variation of the grey level voxel pairs  **From the neighborhood gray-level different matrix (NGLDM):** The NGLDM matrix corresponds to the difference of grey level between one voxel and its 26 neighbours in 3 dimensions.  Coarseness: the level of spatial rate of change in intensity  Contrast: is the intensity difference between neighbouring regions  Busyness: is the spatial frequency of changes in intensity  **From the grey level run length matrix (GLRLM):** The GLRLM matrix gives the size of homogeneous runs for each grey level.  The short-run and the long-run Emphasis (SRE and LRE): is the distribution of the short or the long homogeneous runs in an image  The low grey level and high grey level run emphasis (LGRE and HGRE): is the distribution of the low and high grey level runs.  The short run low and high grey level emphasis (SRLGE and SRHGE): is the distribution of the short homogeneous runs with low or high grey levels.  The long run low and high grey level emphasis (LRLGE and LRHDE): is the distribution of the long homogeneous runs with low or high grey levels  The grey level non uniformity (GLNU): is the non uniformity of the grey levels of the homogeneous runs  The run length non uniformity (RLNU): is the non uniformity of the run lenghts of the homogeneous runs  The run percentage (RP): measured the homogeneity of the homogeneous runs  **From the grey level zone length matrix (GLZLM):** this matrix provides information on the size of the homogeneous zones for each grey level in 3 dimensions.  The short and long zone emphasis (SZE and LZE): is the distribution of the short or the long homogeneous zones in an image.  The log or high grey level zone emphasis (LGZE and HGZE): is the distribution of the low or high grey level zones  The short zone low or high grey level emphasis (SZLGE and SZHGE): is the distribution of the short homogeneous zones with low or high grey levels.  The long zone low or high grey level emphasis (LZLGE and LZHGE): is the distribution of the long homogeneous zones with low or high grey levels.  The grey level non uniformity for zone (GLNU): is the non uniformity of the grey levels of the homogeneous zones  The zone length non uniformity (ZLNU): is the non uniformity of the length of the homogeneous zones  The zone percentage (ZP): measures the homogeneity of the homogeneous zones |
| --- |
